# Variations in the buccal-lingual alveolar bone thickness of impacted mandibular third molar: our classification and treatment perspectives

**DOI:** 10.1038/srep16375

**Published:** 2016-01-13

**Authors:** Jing Ge, Jia-Wei Zheng, Chi Yang, Wen-Tao Qian

**Affiliations:** 1 Department of Oral Surgery, Shanghai Ninth People’s Hospital, College of Stomatology, Shanghai Jiao Tong University School of Medicine, Shanghai Key Laboratory of Stomatology, Shanghai, 200000, China.; 2 Department of Oral-maxillofacial Head and Neck Surgery, Shanghai Ninth People’s Hospital, College of Stomatology, Shanghai Jiao Tong University School of Medicine, Shanghai Key Laboratory of Stomatology, Shanghai, 200000, China.

## Abstract

Selecting either buccal or lingual approach for the mandibular third molar surgical extraction has been an intense debate for years. The aim of this observational retrospective study was to classify the molar based on the proximity to the external cortical bone, and analyze the position of inferior alveolar canal (IAC) of each type. Cone-beam CT (CBCT) data of 110 deeply impacted mandibular third molars from 91 consecutive patients were analyzed. A new classification based on the mean deduction value (MD) of buccal-lingual alveolar bone thickness was proposed: MD≥1 mm was classified as buccal position, 1 mm>MD>−1 mm was classified as central position, MD≤−1 mm was classified as lingual position. The study samples were distributed as: buccal position (1.8%) in 2 subjects, central position (10.9%) in 12 and lingual position (87.3%) in 96. Ninety-six molars (87.3%) contacted the IAC. The buccal and inferior IAC course were the most common types in impacted third molar, especially in lingually positioned ones. Our study suggested that amongst deeply impacted mandibular third molars, lingual position occupies the largest proportion, followed by the central, and then the buccal type.

Although the surgical extraction of impacted mandibular third molar is a common surgical procedure, it still remains a challenge in some complicated cases. Several classifications have been developed aiming at assessing the difficulty of surgical procedure, helping to set up an optimal treatment plan and minimize the incidence of complications. Based on the two-dimensional (2D) radiographic images, there are several classifications. The predominant ones were the Pell-Gregory classification and Winter’s classification^1^. The Pell and Gregory classification considers classes I, II, and III and level A, B, and C based on the position of the mandibular third molar with respect to the mandibular ramus and occlusal plane of the second molar. Winter’s system classified the third molars based on the inclinations of the dental longitudinal axis and occlusal plane, so that the third molar can be: mesio-inclined, vertical or normally inclined, disto-inclined, horizontal, inverted. Furthermore, Pell-Gregory classification has been proved unreliable as a predictor of difficulty in extracting impacted lower third molar^2^; accordingly Pederson proposed a modification of the Pell–Gregory scale that included a third factor, the angulation of the molar (mesioangular, horizontal, vertical, or distoangular). The Pederson scale is widely applied in the field of oral and maxillofacial surgery as a useful way of predicting the surgical difficulty of extraction of impacted lower third molars. However, the mentioned above three classifications were based on the 2D images, and proved to be not fully predictable of the surgical difficulty and less valuable in guiding the clinical extraction procedure^3^. According to nature of overlying tissue, the impacted mandibular third molars were classified as soft tissue impaction, partial bony impaction and fully bony impaction^4^. This system is used by most dental insurance companies and one by which surgeon charges for his services.

As the emerging of three-dimensional (3D) tomography, more and more classifications were proposed based on the 3D image. The morphological shape of the mandible at the third molar region was classified as: round shape (round shape on both buccal and lingual sides), lingual extended (slightly straight on the buccal side with a bony extension on the lingual side), and lingual concave (lingual concave on the lingual side and a round buccal side). This classification aimed at protecting against lingual perforation during mandibular third molar extraction and guiding implant operation^5^,^6^,^7^. The 3D relation between the mandibular third molar and the inferior alveolar canal (IAC) has got increase attention in recent years^8^,^9^,^10^,^11^,^12^,^13^. Predictive variables of classifications were defined as cortication status of IAC, IAC position and IAC shape. Based on the panoramic radiograph, cortication status of IAC with superimposition with the third molar were classified as darkening of roots, deflection of roots, narrowing of roots, bifid root apex, diversion of canal, narrowing of canal, interruption in white line of canal^14^. Based on the 3D tomography imaging, IAC position was classified into five categories: no contact, buccal, lingual, inferior and interradicular^10^,^15^. IAC shape was classified into three categories: round/oval, dumbbell and tear drop^16^.

The key point of the extraction of the impacted lower third molar is the removal of resistant alveolar bone. Knowledge of the alveolar bone thickness in various regions can guide clinicians in deciding the appropriate approach and the proper extraction protocol. Although various classifications exist in literatures, none of those address the buccal and lingual alveolar bone thickness of the impacted third molar. The purpose of this study was to introduce a new classification of impacted mandibular third molars based on buccal and lingual alveolar bone thickness, which were measured on cone-beam computed tomography (CBCT) scans, and present the treatment perspectives based on this classification. The authors hypothesized that this unique classification could classify deeply or fully impacted mandibular third molars based on the proximity to the external cortical bone. The specific aim of the study was to evaluate deeply or fully impacted mandibular third molars’ alveolar bone thickness, analyze the distribution of position type and the IAC position of each type.

## Materials and Methods

### Study design and sample

To address the research purpose, an observational retrospective study was designed and implemented. The study population was composed of CBCT data of 110 consecutive mandibular third molars from the database of Department of Dental Radiology, Ninth People’s Hospital from January 2014 to June 2014. To be included in the study sample, the third molar must be deeply or fully impacted (the impacted tooth is below the cervical line of the adjacent second molar), and the root have been fully developed.

An impacted mandibular third molar was excluded as study subject if it was accompanied with cyst, fracture or tumor, or its CBCT scan images were too vague to measure. The beam-hardening effect was reduced by excluding subjects with dental bridges, dental implants and metal crowns.

The institutional review board and the administrators of the Department of Dental Radiology’s database approved this study. The retrospective study followed the tenets of the Declaration of Helsinki for research involving human subjects, informed consent was obtained from all participants.

### Study variables

The predictor variable was the CBCT data of 110 consecutive mandibular third molars.

CBCT examination was performed with 3D Multi-Image CT (Morita Corp., Japan). The impacted third molars were imaged at a tube voltage of 80 kV, a tube current of 5 mA, an exposure time of 20s, and a voxel size of 0.125 mm. After scanning the contiguous sectional images in three directions: parallel section (parallel to the dental arch), cross-section (perpendicular to the dental arch), and horizontal section (parallel to the occlusal plane), the images were reconstructed from the projection data with a slice thickness of 1 mm.

Two senior oral and maxillofacial surgeons independently evaluated the images in each section on a 17-inch PC monitor. Alveolar bone thickness measurements from the CBCT images were carried out by iDexil software (iDexil Data Viewer, Version 1.27, Morita Corp., Japan). A total of 110 subjects were chosen by using a random table and were measured for 2 times, then remeasured at a 2-week interval for intra- and inter- reliability estimate.

The position of the IAC were observed on reconstructed cross-sectional sections, and assessed by the same 2 surgeons. When there was a disagreement between the 2 surgeons, consensus was reached by discussion.

### Measurement procedure

**Figure 1:**
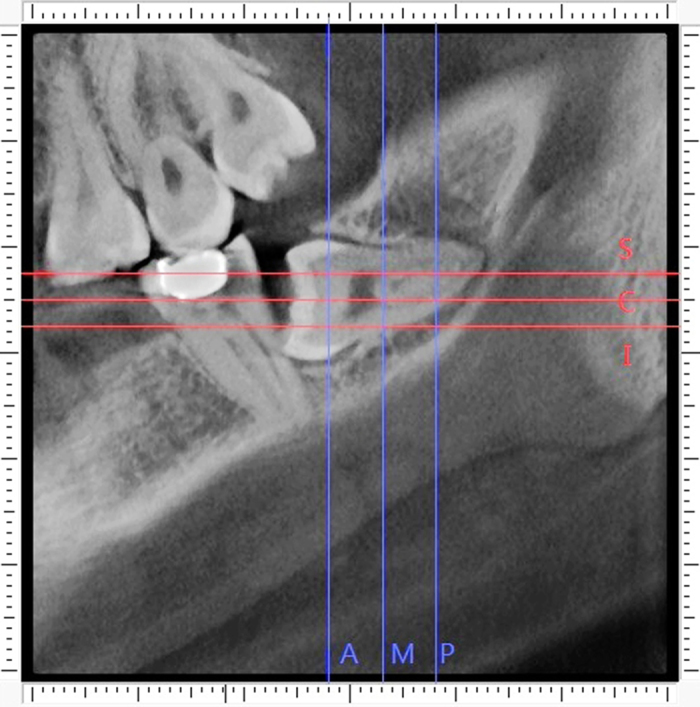
The reference lines. The anterior dividing line (A), the middle dividing line (M) and the posterior dividing line (P) quartered the distance between the anterior and posterior border of the molar. The superior dividing line (S), the central dividing line (C) and the inferior dividing line (I) quartered the distance between the superior and inferior border of the molar.

**Figure 2:**
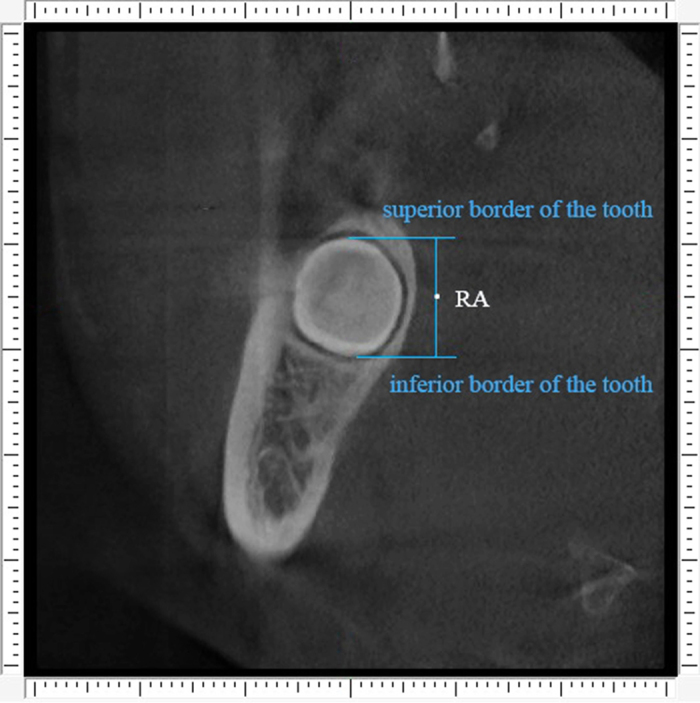
The reference points. Point “RA” represent the middle site of the distance between superior and inferior border of the tooth on the anterior cross section.

**Figure 3:**
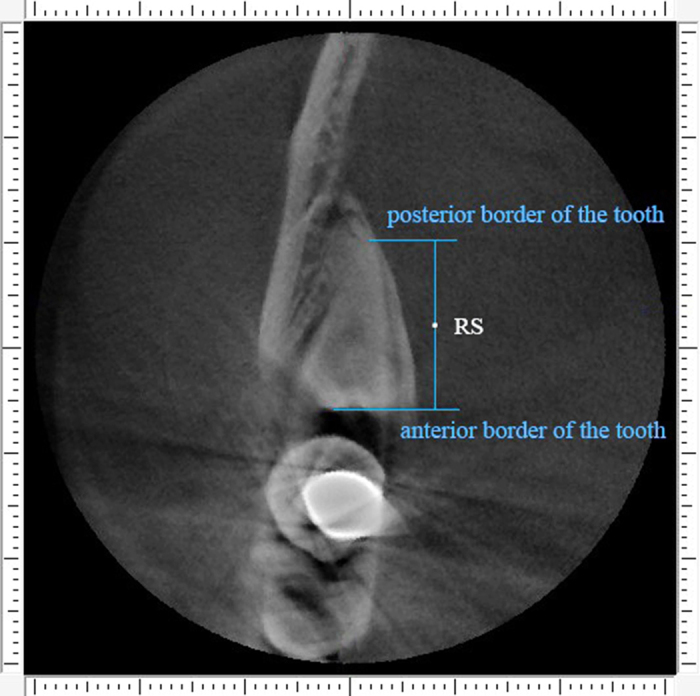
The reference points. Point “RS” represent the middle site of the distance between anterior and posterior border of the tooth on the superior horizontal section.

**Figure 4:**
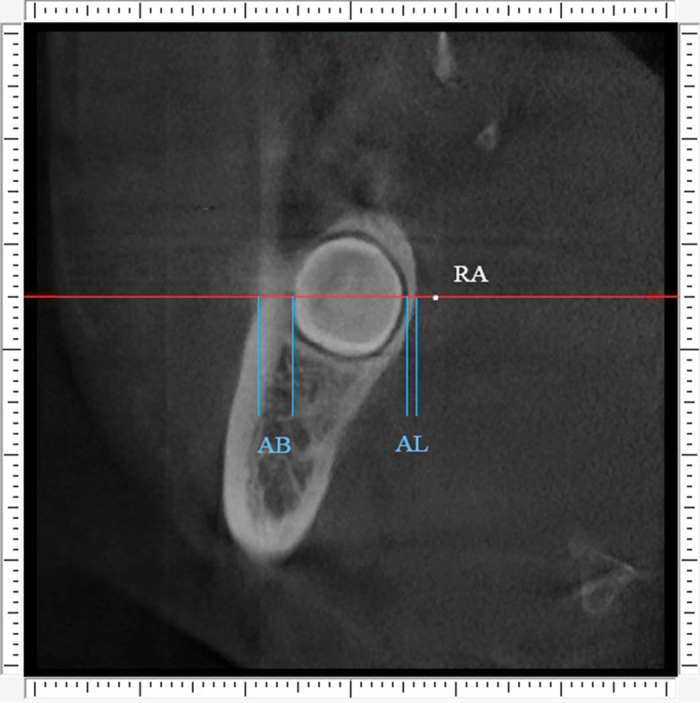
Buccal alveolar bone thickness (AB) and lingual alveolar bone thickness (AL) were measured along the red line on the anterior cross section.

**Figure 5:**
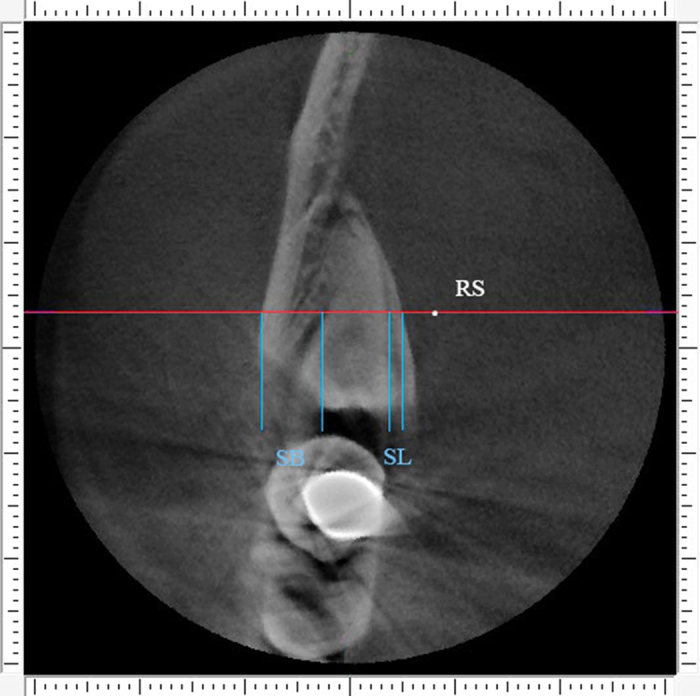
Buccal alveolar bone thickness (SB) and lingual alveolar bone thickness (SL) were measured along the red line on the superior horizontal section.

The detailed procedure of alveolar bone measurement was illustrated in Figs 1, 2, 3, 4, 5.

The first step was to define the sections of reference. The anterior and posterior points of the third molar were located on the parallel section. The distance between the anterior and posterior points was quartered, and the three dividing cross sections were considered as landmarks of measurement and were identified as anterior (A), middle (M) and posterior (P), as blue lines on the Fig. 1. Similarly, the tooth was divided into equal quarters based on the distance between the superior and inferior points of the tooth, and the three dividing horizontal sections were considered as landmarks of measurement and were identified as superior (S), central (C) and inferior (I), red lines as shown on the Fig. 1.

Second step was to define the points of reference. The middle points of the distance between superior and inferior border of the tooth on the anterior, middle and posterior cross sections were located, and were defined as RA (Fig. 2), RM and RP, respectively. The middle points of the distance between anterior and posterior borders of the tooth on the superior, central and inferior horizontal sections were located, and were named RS (Fig. 3), RC and RI, respectively.

Third step was measurement of alveolar bone thickness. For the anterior buccal and lingual bone thickness, we turn to anterior cross section for measurement. The red horizontal line was located through the point “RA”. Two variables were measured along the red horizontal line: “B” was buccal alveolar bone thickness (distance between the outer and inner borders of the buccal plate) and “L” was lingual alveolar bone thickness (distance between the outer and inner borders of the lingual plate). Measurements were made by using the function “slice position” in the software. The outcome variables were termed as AB (the anterior buccal alveolar bone thickness) and AL (the anterior lingual alveolar bone thickness), which were shown on Fig. 4. The same measurement was conducted on the middle and posterior cross section for the value of MB, ML, PB and PL. For the superior buccal and lingual bone thickness, we turn to superior horizontal section for measurement. The red coronal line was located through the point “RS”. Two variables were measured along the red coronal line: “B” was buccal alveolar bone thickness (distance between the outer and inner borders of the buccal plate) and “L” was lingual alveolar bone thickness (distance between the outer and inner borders of the lingual plate). Measurements were made by using the function “slice position” in the software. The outcome variables were termed as SB (the superior buccal alveolar bone thickness) and SL (the superior lingual alveolar bone thickness), which were shown on Fig. 5. The same measurement was conducted on the central and inferior horizontal section for the value of CB, CL, IB and IL.

The total buccal alveolar bone thickness (TB) of the 6 reference points was defined as (AB + MB + PB + SB + CB + IB), and the total lingual alveolar bone thickness (TL) of the 6 reference points was defined as (AL + ML + PL + SL + CL + IL). The mean deduction value of the buccal-lingual alveolar bone thickness (MD) was defined as {(TB–TL)/6}.

**Figure 6:**
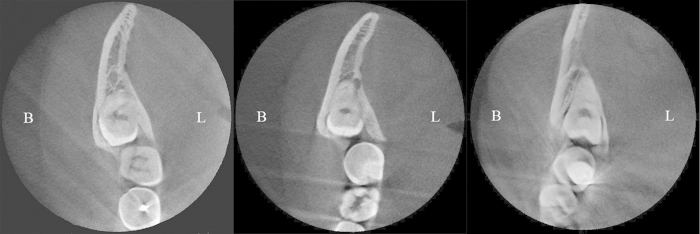
Classification of mandibular third molar’s position based on buccal-lingual alveolar bone thickness, as seen on CBCT parallel sections. (**A**) Buccal position. (**B**) Central position. (**C**) Lingual position.

On the basis of MD value, a new classification of impacted mandibular third molar was proposed as Table 1, and the typical molars of each position type were show on Fig. 6.

### Outcome variables and their assessment

The primary outcome variables were the buccal/lingual alveolar bone thickness (mean ± SD), the mean deduction of the buccal-lingual alveolar bone thickness (MD), the distribution of position type, buccal/lingual alveolar bone thickness (mean ± SD) for each position type, and the IAC position of each type. The third category of variables were collected: age, gender, anatomic position of molar.

### Data analysis

Intra-observer and inter-observer reliability was evaluated using the intra-class correlation coefficient (ICC) by standard statistical software packages (SPSS, version 17.0, Chicago). Intra-observer analysis was based on the MD value, and inter-observer analysis was based on the average MD value of the 2 measurements from each observer. A ICC value < 0.40 was considered poor agreement, 0.40–0.60 was fair agreement, 0.61–0.80 was good agreement and >0.80 was excellent agreement.

The data were input into an Excel spreadsheet, and the final MD value of each measurement was the result of the average of the 4 sets of measurements. According to the MD value, the subjects were classified into three position types. Final data were analyzed using Descriptive statistics.

## Results

In total, 110 molars’ CBCT data from 91 consecutive patients fulfilled the inclusion criteria. There were 48 males and 43 females, aged from 17 to 65 years (mean age of 33 years). Fifty-one of the third molars were on the right side and 59 on the left.

The ICC for inter-observer agreement was about 0.82, demonstrating a good reliability between the observers. The range of ICCs for intra-observer agreement was between 0.94 and 0.98, demonstrating an excellent reliability within the raters.

The buccal and lingual alveolar bone thickness, the distribution of each position type and the IAC position of each type were shown on Table 2.

## Discussion

The purpose of this study was to describe a new classification based on buccal-lingual alveolar bone thickness of the impacted mandibular third molar. The authors hypothesized that the new classification could classify deeply or fully impacted mandibular third molars based on their proximity to the external cortical bone. The specific aim of this study was to measure the deeply or fully impacted mandibular third molars’ alveolar bone thickness and analyze the distribution of each position type. The hypothesis that the new classification could classify deeply or fully impacted mandibular third molars was accepted.

To our knowledge, it is the first time that a new classification based on buccal-lingual alveolar bone thickness of the impacted mandibular third molar is proposed. According to this study: ①the buccal alveolar bone is thicker than the lingual alveolar bone in third molar region; ②lingual position type constitutes the majority of the impacted mandibular third molars, followed by the central position and the buccal type ranks the third. ③The buccal and inferior IAC course were the most prevalent types in impacted third molar, especially in lingually positioned ones.

Deeply or fully impacted mandibular third molars comprise 13.53% of the impacted lower third molars^17^, and their extraction always require removal of large amount of alveolar bone and is prone to develop complications. Diagnosis and treatment of complicated impacted mandibular third molar require comprehensive preoperative examination of the osseous and soft tissue landmarks^18^,^19^. Two-dimensional radiography is far away from adequately reflecting the landmarks of the mandibular third molar^20^. The introduction of CBCT for oral and maxillofacial imaging provides 3D images with lower dose, lower cost and higher spatial resolution than conventional CT^21^,^22^. CBCT is indispensable for optimal risk assessment and adequate surgical planning for complicated impacted mandibular third molar^11^,^18^,^19^,^23^. In addition, CBCT can be used to quantitatively assess alveolar bone thickness with high precision and accuracy^24^. As lower CBCT voxel size can improve the accuracy of alveolar bone linear measurement^25^, high-accuracy CBCT (with a voxel size of 0.125 mm) was adopted in this study to assure the precision of measurement. As the molar’s parallel projection area to buccal and lingual side were the same, the buccal and lingual bone thickness can represent the volume of buccal and lingual alveolar bone. In this study, equally distributed 6 sites were selected for linear measurement, and their mean values represented the bone thickness.

A brand new classification for impacted mandibular third molar was introduced in this study. Consecutive subjects’ data were selected from the database of Department of Dental Radiology, in order to include molars from all departments in our hospital (Department of Oral Surgery, Department of Endodontics, Department of Orthodontics, Department of Prosthodontics, *et al.*), and to avoid selection bias. The results showed that in general, buccal alveolar bone is thicker than lingual alveolar bone in the third molar region. The underlying reason might be the buccal plate is strengthened by the external oblique ridge^26^. The results also showed that the lingual position type is the most common type of impaction patterns, consists 87.3% of the subjects. If applying conventional buccal technique for lingually positioned impacted third molar, often a wide buccal osteotomy is needed. Removing tremendous amount of bone could not only increase the operation time and surgical trauma, but also let the mandible susceptible to intra or postoperative mandibular fracture due to the partial interruption of structural continuity and local weakness resulting from tooth extraction. Furthermore, as the lingual alveolar bone thickness was 1.54 ± 0.59 mm in lingually positioned third molar, preserving of the weak lingual cortex will make it at risk of fracture during tooth luxation, with higher risk of lingual nerve injury and tooth displacement, especially when the lingual plate is perforated^27^. This finding is consistent with that of M. A. Momin *et al.*^5^, whom measured the thinnest part of lingual cortical bone and found that the width was 0.68 mm (ranges from 0.44 to 0.74 mm) in the third molar region.

As inferior alveolar nerve (IAN) injury occurs in approximately 20% to 30% of the cases where a contact relationship is observed between the IAC and mandibular third molar^28^, the position of IAC should always be taken into consideration when designing the surgical approach^11^. The rate of IAC direct contact to the mandibular third molar was 46.7%^20^, and increased to 71.5% when the third molar was impacted^7^. In this study, the rate of IAC direct contact to the deeply or fully impacted mandibular third molar was 87.3%, which makes the analysis of IAC position very important. Although several studies have suggested that the lingual course of the IAC is more common than the buccal course^7^,^23^,^29^,^30^, our findings were in agreement with other studies^10^,^11^,^15^,^16^,^19^,^20^,^31^,^32^,^33^,^34^ that the buccal course is more common than the lingual course, especially in lingually positioned molars (Table 2). Of the molars whose root was in close relationship with the IAC, there was a significant increase of IAN impairment of the lingual IAN course^10^,^11^,^29^,^30^,^34^,^35^. This phenomenon is due to the cortical integrity of the mandibular canal was more likely to be lost when the inferior alveolar nerve was located at the lingual side^35^ and the compression of root when performing the buccal approach^10^,^23^. Ghaeminia *et al.*^23^ suggested that when the IAC was lingual course, the third molar should be luxated in a lingual direction, thereby rotating the apex into a buccal direction to avoid the compression injury. This background justified the choice of lingual approach for the lingually positioned impacted third molars with lingual IAN course. For the lingually positioned impacted third molars with buccal IAN course, the surgeon can remove sufficient lingual bone without fear of IAN damage, and deliver the molar in an lingual direction without any compression to the IAN.

Therefore, treatment perspectives based on the position pattern was proposed: lingual split technique is recommended for lingually positioned impacted mandibular third molar as it might minimize the surgical trauma, improve the surgical efficiency and reduce the incidence of complications; Buccally positioned mandibular third molar is the absolute indication of buccal approach; As for centrally positioned lower third molar, the surgical approach is flexible, depending on individual situation. Eighty-seven deeply or fully impacted mandibular third molars in 72 patients had been successfully extracted according to the treatment perspectives in our department (unpublished data), and a retrospective study investigating the efficiency and safety of the treatment perspectives is currently underway.

Simplified linear measurement was adopted in this study, as its accessibility and convenience make it meaningful for the surgical approach design. Although our measurement method employs basic geometric principles, a number of confounders may have influenced the outcome measures. Considering finite element analysis is gold standard theoretically, sensitivity and specificity of simplified linear measurement compared to the finite element analysis should be addressed in a further study. Nevertheless, using our measurement as a surrogate is still complicated and time-consuming for daily clinical practice. Visual observation is recommended for obvious buccally or lingually positioned molars, especially for experienced surgeons. Moreover, in order to validate the treatment principle proposed in this study, further research could be designed as a prospective randomized controlled trial to compare the effect of different surgical approaches used in impacted mandibular third molars’ removal of different position pattern. However, the choice of the surgical access should always be reached through a careful clinical and radiographic diagnosis, including the root number and shape, the remaining mandible height, as well as relationship with adjacent second molar^36^,^37^.

## Conclusions

From the results of the study, it can be concluded that amongst deeply or fully impacted mandibular third molars, the lingual position type occupies the largest proportion; the central position type ranks the second and followed by the buccal position type. Buccal and inferior IAC course are the most common types in the lingually positioned impacted molar. Lingual split technique is recommended for lingually positioned impacted mandibular third molar. Buccally positioned mandibular third molar is the absolute indication of buccal approach. As for centrally positioned lower third molar, the surgical approach is flexible, depending on the individual variation.

## Additional Information

**How to cite this article**: Ge, J. *et al.* Variations in the buccal-lingual alveolar bone thickness of impacted mandibular third molar: our classification and treatment perspectives. *Sci. Rep.* **6**, 16375; doi: 10.1038/srep16375 (2016).

## Figures and Tables

**Table 1:** A classification of impacted mandibular third molar.

Classification	MD = (Total buccal bone thickness - Total lingual bonethickness)/6
Buccal position	MD ≥ 1 mm
Central position	−1 mm < MD < 1 mm
Lingual position	MD ≤ −1 mm

**Table 2:** The distribution, bone thickness and IAC position of each position type.

Classification		Buccal position	Central position	Lingual position	Total
	Number (percentage)	2 (1.8%)	12 (10.9%)	96 (87.3%)	110
	Buccal bone thickness (mm)	3.76 ± 0.13	2.82 ± 0.71	4.76 ± 1.16	4.51 ± 1.30
	Lingual bone thickness (mm)	2.13 ± 0.23	2.58 ± 0.65	1.54 ± 0.59	1.69 ± 0.73
IAC position	No contact, n(%)	0 (0%)	1 (8.3%)	13 (13.5%)	14 (12.7%)
	Buccal, n(%)	0 (0%)	2 (16.7%)	35 (36.5%)	37 (33.6%)
	Lingual, n(%)	1 (50%)	2 (16.7%)	14 (14.6%)	17 (15.5%)
	Inferior, n(%)	1 (50%)	6 (50%)	32 (33.3%)	39 (35.5%)
	Interradicular, n(%)	0 (0%)	1 (8.3%)	2 (2.1%)	3 (2.7%)
